# Auriculotemporal Frey syndrome not associated with surgery or diabetes: systematic review

**DOI:** 10.1007/s00431-022-04415-w

**Published:** 2022-02-19

**Authors:** Céline Betti, Gregorio P. Milani, Sebastiano A. G. Lava, Mario G. Bianchetti, Gabriel Bronz, Gian P. Ramelli, Barbara Goeggel Simonetti, Marcel M. Bergmann

**Affiliations:** 1 Pediatric Institute of Southern Switzerland, Ente Ospedaliero Cantonale, Ospedale San Giovanni, Bellinzona, Switzerland; 2 Pediatric Unit, Fondazione IRCCS Ca’ Granda Ospedale Maggiore Policlinico, Milan, Italy; 3 Department of Clinical Sciences and Community Health, Università degli Studi di Milano, Milan, Italy; 4 Pediatric Cardiology Unit, Department of Pediatrics, Centre Hospitalier Universitaire Vaudois and University of Lausanne, Lausanne, Switzerland; 5 Heart Failure and Transplantation, Department of Pediatric Cardiology, Great Ormond Street Hospital, London, UK; 6 Family Medicine, Faculty of Biomedical Sciences, Università della Svizzera Italiana, Lugano, Switzerland; 7 Faculty of Biomedical Sciences, Università della Svizzera Italiana, Lugano, Switzerland; 8 Department of Neurology, University Hospital Bern, University of Bern, Bern, Switzerland; 9 Centro Pediatrico del Mendrisiotto, Mendrisio, Switzerland; 10 Pediatric Allergy Unit, Department of Woman, Child and Adolescent, University Hospitals of Geneva, Geneva, Switzerland

**Keywords:** Auriculotemporal syndrome, Forceps birth, Frey syndrome, Gustatory sweating, Pseudo-allergic reaction

## Abstract

Patients who undergo salivary gland, neck, or facelift surgery or suffer from diabetes mellitus often develop Frey syndrome (also known as auriculotemporal syndrome or gustatory sweating). Frey syndrome has been occasionally reported to occur in subjects without history of surgery or diabetes but this variant of Frey syndrome has not been systematically investigated. We searched for original articles of Frey syndrome unrelated to surgery or diabetes without date and language restriction. Article selection and data extraction were performed in duplicate. Our systematic review included 76 reports describing 121 individual cases (67 males and 54 females) of Frey syndrome not associated with surgery or diabetes. The age at onset of symptoms was ≤ 18 years in 113 (93%) cases. The time to diagnosis was 12 months or more in 55 (45%) cases. On the other hand, an allergy evaluation was performed in half of the cases. A possible cause for Frey syndrome was detected in 85 (70%) cases, most frequently history of forceps birth (*N* = 63; 52%). The majority of the remaining 22 cases occurred after a blunt face trauma, following an auriculotemporal nerve neuritis or in association with a neurocutaneous syndrome. The cause underlying Frey syndrome was unknown in 36 cases.

*   Conclusion*: Frey syndrome not associated with surgery or diabetes almost exclusively affects subjects in pediatric age and is uncommon and underrecognized. Most cases occur after forceps birth. There is a need to expand awareness of this pseudo-allergic reaction among pediatricians and allergists.

**Table:** 

**What is Known:** *• Pre-auricular reddening, sweating, and warmth in response to mastication or a salivary stimulus characterize Frey syndrome.* *• It usually occurs after salivary gland surgery and in diabetes.*	
**What is New:** *• In children, Frey syndrome is rare, and most cases occur after a forceps-assisted birth.* *• In childhood, this condition is often erroneously attributed to food allergy.*

**What is Known:**

*• Pre-auricular reddening, sweating, and warmth in response to mastication or a salivary stimulus characterize Frey syndrome.*

*• It usually occurs after salivary gland surgery and in diabetes.*

**What is New:**

*• In children, Frey syndrome is rare, and most cases occur after a forceps-assisted birth.*

*• In childhood, this condition is often erroneously attributed to food allergy.*

## Introduction

Mastication or a salivary stimulus may be followed by rapid reddening of the pre-auricular region, normally associated with local warmth, sweating, and general discomfort, and occasionally also with shedding of tears or watery otorrhea [1]. This phenomenon was first described by M. Duphenix in 1757 and subsequently by J. Baillarger in 1853 [2, 3]. However, the better characterization was made in 1923 by the Polish Jewish neurologist Łucja Frey-Gottesman (1889–1942). The condition is nowadays referred to as auriculotemporal (nerve) syndrome, gustatory sweating, or Frey syndrome [2, 3]. It typically and rather commonly occurs 6 to 18 months after salivary gland, neck, or facelift surgery [1], and in diabetes mellitus [4].

The aims of this systematic review of the literature were to document demographics, presentation, diagnostic difficulties, and causes of Frey syndrome not associated with surgery or diabetes.

## Methods

### Literature search strategy

The PRISMA recommendations for reporting systematic reviews were used. The literature search was conducted in the databases Excerpta Medica, National Library of Medicine, and Web of Science up to July 2021 without date and language restriction. Search terms were “auriculotemporal syndrome”, “gustatory sweating”, or “Frey syndrome”. References listed within bibliographies of the retrieved records and reports already known to the authors were also considered for inclusion.

The literature search was conducted by two investigators, who independently screened titles and abstracts of all reports in a non-blinded fashion to remove irrelevant reports. Discrepancies in study identification were adjudicated by a senior investigator. Subsequently, full-text publications were reviewed to decide whether the report fitted the eligibility criteria of the review.

### Selection criteria—data extraction

Original articles reporting individual cases of Frey syndrome not associated with surgery or diabetes were considered. Only patients with episodes of (a) acute onset of reddening of the pre-auricular region (with or without local warmth, general discomfort, sweating, shedding of tears, or watery otorrhea), (b) beginning within a few seconds after onset of eating (or even immediately before eating), and (c) resolving within minutes after termination were included. The history and the examination of each case were carefully addressed with respect to laterality, age at symptoms onset, time interval from symptoms onset to diagnosis, past medical history including forceps-assisted vaginal birth [5], face trauma, coexisting conditions, or evaluation by an allergic disease specialist (with or without testing for immediate-type hypersensitivity). The occurrence of a positive family history, defined as at least two first-degree family members with Frey syndrome not associated with surgery or diabetes, was also addressed. If needed, attempts were made to contact original authors to obtain missing information.

### Completeness of reporting—analysis

Completeness of reporting was judged for each included case using the following three components [6]: (1) detailed description of history, symptoms, and findings; (2) information on diagnostic workup, and (3) management and follow-up. Each component was rated as 1, 2, or 3 and the reporting quality was graded according to the sum of each item as excellent (7 to 9), good (4 to 6), or acceptable (≤ 3).

Categorical data are presented as counts and were analyzed using the Fisher exact test [7]. Continuous data are shown as medians and interquartile ranges and were compared using the Kruskal–Wallis test with the post hoc Dunn comparison [7]. Statistical significance was defined by two-sided *P*-values of < 0.05.

## Results

### Search results—reporting completeness

**Fig. 1:**
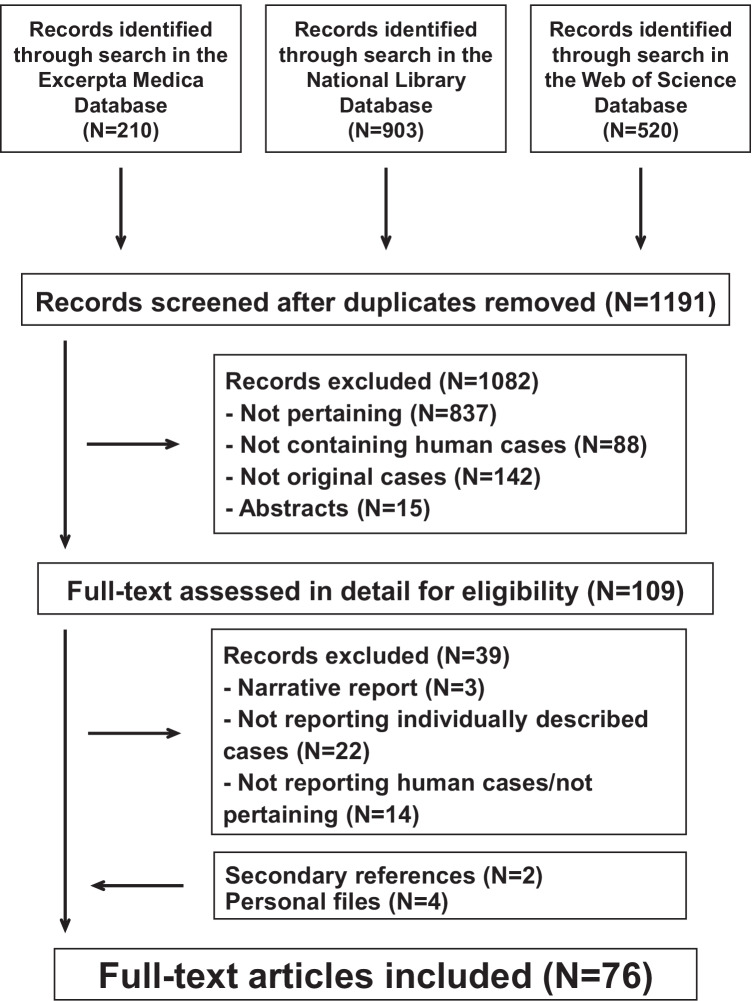
Auriculotemporal Frey syndrome not associated with surgery or diabetes mellitus. Flowchart of the literature search process

The literature search returned 1191 potentially relevant reports (Fig. 1). After removing irrelevant reports, 109 full-text publications were reviewed for eligibility. For the final analysis, we retained 76 reports describing 121 individual cases of Frey syndrome not associated with surgery or diabetes [5, 8–82]. The mentioned reports were published since 1945 in English (*N* = 54), Spanish (*N* = 20), and French (*N* = 2) from the following countries: Spain (*N* = 23), the USA (*N* = 15), the UK (*N* = 13), France (*N* = 3), Canada (*N* = 3), Turkey (*N* = 3), Brazil (*N* = 2), Germany (*N* = 2), Israel (*N* = 2), Australia (*N* = 1), Austria (*N* = 1), Belgium (*N* = 1), Colombia (*N* = 1), Denmark (*N* = 1), Dubai (*N* = 1), India (*N* = 1), Ireland (*N* = 1), Italy (*N* = 1), and South Africa (*N* = 1). Reporting comprehensiveness was excellent in 21, good in 92, and acceptable in the remaining 8 cases.

### Search results

#### Presentation

In the 121 cases, age at onset of symptoms was ≤ 18 years in 113 cases (93%). A reddening of the pre-auricular region was observed in all cases. Sweating, local warmth, and general discomfort were reported only in a minority of cases and were significantly less common among pediatric cases (Table 1). Shedding of tears or watery otorrhea was never noted. Wheals, itching, scratch marks, and symptoms or signs consistent with a multisystem involvement were also never reported.

**Table 1:** Symptoms and signs other than local reddening in 121 cases of auriculotemporal Frey syndrome without history of salivary gland, neck, or facelift surgery

	**All**	**Children**	**Adults**	***P*-value**
*N*	121	113	8	
Sweating, *N*	14	6	8	< 0.01
Local warmth, *N*	13	6	7	< 0.01
General discomfort, *N*	6	0	6	< 0.01
Shedding of tears, *N*	0	0	0	
Watery otorrhea, *N*	0	0	0	

Frey syndrome was unilateral in 100 (83%) cases (right-sided more frequently than left-sided) and bilateral in the remaining 21 (17%) cases (Table 2). The time to diagnosis was ≥ 12 months in 55 (45%) cases. On the other hand, an allergy evaluation was performed in half of the cases. A possible cause for Frey syndrome was detected in 85 (70%) cases, most frequently history [9, 10, 12, 14, 15, 18, 20, 22, 23, 26, 28, 30–34, 36, 38, 41, 42, 49, 50, 54, 57, 58, 62, 63, 66–68, 71, 79, 82, 83] of forceps-assisted birth (*N* = 63; 52%). The majority of the remaining 22 cases occurred after a blunt face trauma, following an auriculotemporal nerve neuritis or in association with a neurocutaneous syndrome (Table 3) and were significantly older (*P* < 0.05) than the other cases. The cause underlying Frey syndrome was unknown in the remaining 36 cases. Interestingly, Frey syndrome was bilateral in 15 (41%) of the latter 36 cases (*P* < 0.0001 versus remaining cases). Furthermore, the cases noted after forceps birth were more frequently male (*P* < 0.05 versus non-forceps cases).

**Table 2:** Characteristics of 121 patients 0.15 to 79 years of age affected by auriculotemporal Frey syndrome without history of salivary gland, neck, or facelift surgery. Date are presented either as frequency or as median and interquartile range

	**All**	**Cause found**		**Unknown causes**^**◆**^
		**Forceps birth**	**Further causes**	
*N*	121	63	22	36
Males to Females	67:54	41:22^✙^	11:11	16:20
Age at onset of symptoms				
Years	0.6 [0.4–1.0]	0.5 [0.4–0.6]	5.8^●^ [1.3–18]	0.5 [0.4–1.2]
≤ 18 years, *N*	113	63	16	34
Time to diagnosis				
Months	7.2 [1.2–24]	7.2 [1.2–36]	6.0 [0.0–16]	3.6 [0.0–17]
≥ 12 months, *N*	55	30	12	13
Familiarity, *N*	7	3	0	4
Laterality				
Unilateral	100	59	20	21
Right	56	36	12	8
Left	38	19	8	11
Unspecified	6	4	0	2
Bilateral	21	4	2	15^**⤬**^
Allergy investigation	60	40	4	16
Without testing	25	16	8	1
With testing	35	24	3	8

^**◆**^Delivery was explicitly non-forceps-assisted in 31 of the 36 cases (no corresponding information was available for the remaining cases). ^✙^*P* < 0.05 versus non-forceps. ^●^*P* < 0.05 versus forceps birth and unknown causes. ^**⤬**^*P* < 0.0001 versus further cases and forceps-assisted birth

**Table 3:** Causes other than forceps-assisted delivery in 22 subjects (11 males and 11 females) with Frey syndrome without history of surgery

**Cause**	**No**	**Age at symptoms onset (years)**	**References**
**Blunt face trauma**			
With mandibular condyle facture	8	2.1, 13, 17, 22, 23, 26, 28, adulthood	[11, 13, 21, 25, 52, 55, 61, 75]
Without mandibular condyle fracture	1	0.4	[14]
**Congenital neurocutaneous syndrome**			
Neurofibromatosis type 1	4	0.4, 0.8, 1.2, 5.8	[51, 53, 80]
PHACE syndrome	1	0.1	[81]
**Auriculotemporal nerve neuritis**			
Herpes simplex virus	2	6.0, 20	[29, 74]
Varicella zoster virus	2	1.2, 6.5	[64, 76]
**Further causes**			
Trifid mandibular condyle	1	Childhood	[60]
Facial burns	1	Childhood	[77]
Parotid hemangiopericytoma	1	1.3	[47]
Moebius syndrome	1	0.7	[73]

### Management—course

Reassurance about the benign and non-allergic nature of symptoms and signs was the most frequently recommended treatment. One adult case [77] was injected intradermally botulinum toxin (but the effect on the symptoms was poorly documented).

The follow-up was documented in 37 cases: (a) all symptoms and signs disappeared within 2 months to 5 years in 14 cases; (b) symptoms and signs ameliorated within 5 weeks to 2½ years in 13 cases; (c) symptoms and signs were unaltered after a follow-up of 4 months to 6 years in the remaining 10 cases.

## Discussion

Frey syndrome mainly results from an aberrant innervation of the auriculotemporal branch of the mandibular nerve following surgery in the proximity of the parotid gland or in the context of diabetes [1, 4]. The present literature review on auriculotemporal Frey syndrome not associated with surgery or diabetes may be essentially recapitulated and discussed in four points.

First, most case cases are unilateral and occur in pediatric age.

Second, three very typical features of Frey syndrome [1], that is, sweating, local warmth, and general discomfort, are relatively uncommon in subjects ≤ 18 years of age.

Third, various causes may underlie this form of Frey syndrome including blunt face trauma, neuritis, and a neurocutaneous syndrome. However, every second case occurs in children with history of forceps birth. Hence, it is assumed that forceps birth occasionally results in Frey syndrome [83]. Finally, no apparent cause was found in about one-third of cases. Many cases of Frey syndrome of unknown causes were bilateral or familial. It is therefore tempting to assume that prenatal factors account for some of the latter cases (the term idiopathic has also been suggested to denote these cases).

Fourth, the diagnosis of Frey syndrome not associated with surgery or diabetes may be tricky, the time to diagnosis is often ≥ 12 months and an allergy evaluation is performed in every second case. In our opinion, in Frey syndrome, many points should divert from suspecting food allergy as the cause, including the reddening occurring in the absence of pruritus, angioedema, signs consistent with a multisystem involvement, the quick spontaneous disappearance, and the occurrence on challenge with a variety of foods, as shown in Table 4 [84].

**Table 4:** Differential diagnosis between auriculotemporal Frey syndrome and immediate type food allergy in infancy and childhood

	**Auriculotemporal Frey syndrome**	**Immediate type food allergy**
Cause	Aberrant innervation of the auriculotemporal nerve	Immune mediated
Culprit food	Any food	Specific food*
Time latency from food intake	≤ 30 s (at times before mastication)	Immediate (occasionally 1–2 h)
Skin involvement		
Localization	Pre-auricular region, mostly unilateral	Any skin area including mucosae
Findings	Erythema, warmth, and occasionally sweating	Urticaria, angioedema
Multisystem involvement	None	Respiratory, cardiovascular, gastrointestinal

^*^Most cases (≥ 90%) are triggered by cow’s milk, hen’s egg, soy, wheat, fish, tree nuts, seeds, peanuts, and shellfish

The results of this systematic review, which included 121 cases, are in line with those of a French multicenter inquiry among pediatricians and allergists that disclosed 48 Frey syndrome cases [85].

Reassurance represents in our opinion the mainstay of management in Frey syndrome not associated with surgery or diabetes [86, 87]. In addition to obtaining a detailed history and examination, reassurance therapy consists of making the diagnosis, explaining the pathophysiology of signs and symptoms, emphasizing that there is nothing to worry about, suggesting that signs and symptoms often ameliorate or resolve with time, discouraging further investigations, eliminating diets or restrictions, and recommending regular follow-up appointments [86, 87]. Intradermic botulinum toxin has been shown to reduce excessive sweating in postsurgical Frey syndrome [1]. Available data do not support the prescription of this treatment option in children with Frey syndrome, mainly because sweating is rare in these cases.

The most important limitation of this review arises from the small number of published reports on Frey syndrome not associated with surgery or diabetes. Furthermore, completeness in reporting cases was often rather low.

## Conclusions

Frey syndrome not associated with surgery or diabetes almost exclusively affects subjects in pediatric age, and is uncommon and underrecognized. Forceps birth is the most frequent cause. The initial diagnosis may be established based on history and physical examination alone. There is a need to expand awareness of this pseudo-allergic reaction among pediatricians and allergists to avoid redundant investigations, unnecessary management, and apprehension.

## Data Availability

Not applicable.

## References

[1] Motz KM, Kim YJ (2016). Auriculotemporal syndrome (Frey syndrome). Otolaryngol Clin North Am.

[2] Dulguerov P, Marchal F, Gysin C (1999). Frey syndrome before Frey: the correct history. Laryngoscope.

[3] Grzybowski A, Sak J (2012). Lucja Frey (1889–1942): life destroyed by the Holocaust - on the 70^th^ anniversary of her death. Clin Dermatol.

[4] Urman JD, Bobrove AM (1999). Diabetic gustatory sweating successfully treated with topical glycopyrrolate: report of a case and review of the literature. Arch Intern Med.

[5] Haxton HA (1948). Gustatory sweating. Brain.

[6] Vismara SA, Lava SAG, Kottanattu L, Simonetti GD, Zgraggen L, Clericetti CM, Bianchetti MG, Milani GP (2020). Lipschütz’s acute vulvar ulcer: a systematic review. Eur J Pediatr.

[7] Brown GW, Hayden GF (1985). Nonparametric methods. Clinical applications. Clin Pediatr.

[8] Anonymous (1945) Unilateral flush after food. Br Med J 210:687–688

[9] Dey DL (1953). A variant of the auriculo-temporal syndrome. Aust N Z J Surg.

[10] Martis C, Athanassiades S (1969). Auriculotemporal syndrome (Frey’s syndrome), secondary to fracture of the mandibular condyle. Plast Reconstr Surg.

[11] Balfour HH, Bloom JE (1970). The auriculotemporal syndrome beginning in infancy. J Pediatr.

[12] Storrs TJ (1974). A variation of the auriculotemporal syndrome. Br J Oral Surg.

[13] Davis RS, Strunk RC (1981). Auriculotemporal syndrome in childhood. Am J Dis Child.

[14] Sly RM (1981). Auriculotemporal syndrome. Cutis.

[15] Beck SA, Burks AW, Woody RC (1989). Auriculotemporal syndrome seen clinically as food allergy. Pediatrics.

[16] De Benedittis G (1990). Auriculotemporal syndrome (Frey’s syndrome) presenting as tic douloureux. Report of two cases J Neurosurg.

[17] Clarós P, González-Enseñat MA, Arimany J, Vincente MA, Clarós A (1993). Síndrome de Frey en la infancia [Frey syndrome in childhood]. Acta Otorrinolaringol Esp.

[18] Kozma C, Gabriel S (1993) Gustatory flushing syndrome. A pediatric case report and review of the literature. Clin Pediatr (Phila) 32:629–631 10.1177/000992289303201013 8261729

[19] Johnson IJ, Birchall JP (1995). Bilateral auriculotemporal syndrome in childhood. Int J Pediatr Otorhinolaryngol.

[20] Mellor TK, Shaw RJ (1996). Frey’s syndrome following fracture of the mandibular condyle: case report and literature review. Injury.

[21] Sicherer SH, Sampson HA (1996). Auriculotemporal syndrome: a masquerader of food allergy. J Allergy Clin Immunol.

[22] Dizon MV, Fischer G, Jopp-McKay A, Treadwell PW, Paller AS (1997) Localized facial flushing in infancy. Auriculotemporal nerve (Frey) syndrome. Arch Dermatol 133:1143–1145 9301592

[23] Cliff S, Lever R, Moss AL, Mortimer PS (1998). Frey’s syndrome without hyperhidrosis. J R Soc Med.

[24] Kaddu S, Smolle J, Komericki P, Kerl H (2000). Auriculotemporal (Frey) syndrome in late childhood: an unusual variant presenting as gustatory flushing mimicking food allergy. Pediatr Dermatol.

[25] Rodriguez-Serna M, Marí JI, Aliaga A (2000). What syndrome is this? Auriculotemporal nerve (Frey) syndrome. Pediatr Dermatol.

[26] Moreno-Arias GA, Grimalt R, Llusa M, Cadavid J, Otal C, Ferrando J (2001). Frey’s syndrome. J Pediatr.

[27] Reche Frutos M, García Ara MC, Boyano T, Díaz Pena JM (2001). Syndrome auriculotemporal Allergol Immunopathol (Madr).

[28] Drummond PD (2002). Mechanism of gustatory flushing in Frey’s syndrome. Clin Auton Res.

[29] Karunananthan CG, Kim HL, Kim JH (2002). An unusual case of bilateral auriculotemporal syndrome presenting to an allergist. Ann Allergy Asthma Immunol.

[30] Labarta N, Olaguibel JM, Gómez BG, Lizaso MT, García BE, Echechipía S, Tabar AI (2002). Síndrome del nervio auriculotemporal. Diagnóstico diferencial con alergia alimentaria [Auriculotemporal nerve syndrome. Differential diagnosis to food allergy]. Alergol Inmunol Clin.

[31] Gonzàlez-Mendiola R, Sánchez-Fernández C, De la Hoz-Caballer B, Prieto-Montaño P, Muñoz-Martín T, Garcia-González MC, Sánchez-Cano M (2003). Auriculotemporal syndrome: differential diagnostic of food allergy. Allergy.

[32] Zacharisen MC, George RA (2003). Recurrent rash associated with food ingestion in a 5-year-old child. Ann Allergy Asthma Immunol.

[33] Ott H, Brost H, Poblete-Gutiérrez P, Schröder CM, Frank J (2004). Auriculotemporal syndrome in childhood. Acta Derm Venereol.

[34] Grouhi M, Alshehri M (2005). Auriculotemporal’s syndrome in three siblings and literature review. Curr Pediatr Res.

[35] Carpintero Hurtado N, Sainz Gómez C, García Cariñena M, Virto Ruiz MT (2006). Síndrome de Frey: tres observaciones clínicas con dos etiopatogenias diferentes [Frey’s syndrome: report of three cases with two distinct etiopathogeneses]. An Pediatr (Barc).

[36] Costa Orvay JA, González Enseñat MA, Vicente Villa MA, Morales Castillo E, Campistol PJ (2006). Síndrome de Frey en la infancia: una enfermedad muy infrecuente [Frey’s syndrome in childhood: a highly infrequent disease]. An Pediatr (Barc).

[37] Dutau G, Goldberg M (2006). Le syndrome de Lucie Frey et ses variantes (syndrome des flushs gustatifs unilatéraux). Revue à propos d’une observation pédiatrique [Frey’s syndrome and its variants (unilateral gustatory sweating). Review, with report of a case in a child]. Rev Fr Allergol Immunol Clin.

[38] Álvarez Cuesta CC, Rodríguez Díaz E, García Bernrdez AM, Galache Osuna C, Blanco Barrios S, Menéndez Fernández JM (2007). Síndrome auriculotemporal de Frey. Un caso de presentación bilateral en un lactante [Frey auriculotemporal syndrome. A bilateral case in a child]. Med Cutan Ibero Lat Am.

[39] Díez E, Boixeda P (2007). Síndrome de Frey en la infancia [Frey’s syndrome in childhood]. Actas Dermosifiliogr.

[40] Escudero-Cantó MC, Cuartero-del Pozo I, Ruiz-Cano R, Balmaseda-Serrano E, Gil-Pons E, Onsurbe I (2007). Síndrome del nervio auriculotemporal en niños secundario a un parto instrumentado con fórceps [Auriculotemporal nerve syndrome in children secondary to a forceps delivery]. Rev Neurol.

[41] Mozes F, Tukiyama FA, Castro APBM, Corradi GA, Pastorino AC, Fomin ABF, Jacob CMA (2007). Síndrome de Frey simulando eritema malar por alergia alimentar [Frey’s syndrome simulating malar flushing by food allergy]. Rev Paul Pediatr.

[42] Fernández Tejada E, Fernández García N, Meana Meana A, López VP (2008). Síndrome auriculotemporal (síndrome de Frey) en dos lactantes con presentación bilateral. Rev Pediatr Aten Primaria.

[43] Lam J, Milligan J (2009). Case 2: unilateral facial flushing with eating. Paediatr Child Health.

[44] Sethuraman G, Mancini AJ (2009). Familial auriculotemporal nerve (Frey) syndrome. Pediatr Dermatol.

[45] Tamayo-Quijano LM, Chinchilla-Mejía CF, Toro-Giraldo AM (2009). Eritema lineal facial en un niño [Linear erythema on the face of a boy]. Actas Dermosifiliogr.

[46] Farman M, Zaitoun H (2010). Auriculotemporal nerve syndrome in association with congenital haemangiopericytoma: a case report. Eur J Paediatr Dent.

[47] Hussain N, Dhanarass M, Whitehouse W (2010). Frey’s syndrome: a masquerader of food allergy. Postgrad Med J.

[48] Madrigual Diaz C (2010). Eritema facial unilateral asociado a estímulos gustativos en un lactante: síndrome de Frey [Unilateral facial erythema in response to gustatory stimuli in an unweaned baby: Frey syndrome]. Acta Pediatr Esp.

[49] Martínez-Baylach J, Aragó T, Galdós H, Herrera C, Rubio de Abajo I (2010) Síndrome de Frey secundario a traumatismo obstétrico. Presentación de 2 casos [Frey’s sydrome secondary to an obstetrics trauma: presentation of 2 cases and a review of the literature]. An Pediatr (Barc) 72:272–277 10.1016/j.anpedi.2009.11.015 20188641

[50] Ibrahim LF, Brenner C, McMenamin J, Webb D (2011). Frey syndrome in neurofibromatosis 1. BMJ Case Rep.

[51] Kragstrup TW, Christensen J, Fejerskov K, Wenzel A (2011). Frey syndrome-an underreported complication to closed treatment of mandibular condyle fracture? Case report and literature review. J Oral Maxillofac Surg.

[52] Listernick R, Legius E, Charrow J (2011). Gustatory flushing (auriculotemporal nerve syndrome) in children with neurofibromatosis type 1 and facial plexiform neurofibromas. J Pediatr.

[53] Suárez Castañón C, Mellado Peña MJ, Joaqui López N, Villota Arrieta J, López-Hortelano G (2011). M. Síndrome de Frey: una entidad a diferenciar de la alergia alimentaria [Frey’s syndrome: an entity to differentiate from food allergy]. Rev Esp Pediatr.

[54] Bulut E, Bekçioğlu B (2012). Delayed Frey syndrome after closed treatment of condylar fracture. J Craniofac Surg.

[55] Martínez BJ (2012). Síndrome de Frey o auriculotemporal en Pediatría: importancia de su conocimiento [Frey’s or auriculotemporal syndrome in childhood: importance of its knowledge]. Form Act Pediatr Aten Prim.

[56] Ortega Casanueva C, Sánchez-García S, Rodríguez del Río P, Escudero C, Andregnette V, Ibáñez MD (2012) Frey syndrome in children: a nonallergic cause of facial erythema triggered by food. J Investig Allergol Clin Immunol 22:295–297 22812203

[57] Caulley L, Hong P (2013). Pediatric auriculotemporal nerve (Frey) syndrome. CMAJ.

[58] Humphrey J, Black G, Wild L (2013). Facial flushing with food: the auriculotemporal syndrome. J Gen Intern Med.

[59] Motta-Junior J, Aita TG, Pereira-Stabile CL, Stabile GA (2013). Congenital Frey’s syndrome associated with nontraumatic bilateral trifid mandibular condyle. Int J Oral Maxillofac Surg.

[60] Demirseren DD, Akoglu G, Emre S, Metin A (2014). A case of Frey’s syndrome. Indian J Dermatol Venereol Leprol.

[61] Dutau G (2014). Le syndrome des flushs gustatifs. Revue critique à propos d’une observation pédiatrique [Frey syndrome and variants (gustatory flushing syndromes): a paediatric case and review of the literature]. Lett ORL Chir Cervicofac.

[62] Giovannini-Chami L, Blanc S, Albertini M, Bourrier T (2014). Frey’s syndrome: differential diagnosis of food allergy. Arch Dis Child.

[63] Bourgeois P, Morren MA (2015). Frey’s syndrome after Herpes Zoster virus infection in a 2-year-old girl. Pediatr Dermatol.

[64] Buyuktiryaki B, Sekerel BE (2015). Is it food allergy or Frey syndrome?. J Allergy Clin Immunol Pract.

[65] Martin FP, Bruning CV, Cox H (2015). Síndrome de Frey en una lactante [Frey syndrome in infancy]. Rev Chil Dermatol.

[66] Tillman BN, Lesperance MM, Brinkmeier JV (2015). Infantile Frey’s syndrome. Int J Pediatr Otorhinolaryngol.

[67] Daniels E, Watchorn R (2016) Unilateral facial flushing precipitated by eating. BMJ 352:i1377 10.1136/bmj.i1377 26960312

[68] Hassan S, Saviour MJ (2016). Recurring facial erythema in an infant. Case Rep Pediatr.

[69] Peinado Adiego C, Olloqui Escalona A, Arcauz Eguren MP (2016). Síndrome de Frey o síndrome auriculotemporal [Frey s syndrome or auriculotemporal syndrome]. Rev Pediatr Aten Primaria.

[70] Álvarez Zallo N, Martínez Ortiz A, García Blanco L, Martínez Ganuza B, Ruiz Goikoetxea M (2017). Síndrome de Frey: a propósito de dos casos [Frey syndrome in children: two case report]. Acta Pediatr Esp.

[71] Baquedano Lobera I, Bernad Fonz R, Marín Andrés M, García Vera C, Arana Navarro T, Cenarro GT (2017). Erupción facial tras ingesta de papilla de frutas [Facial rash after ingestion of fruit porridge]. Bol Pediatr Arag Rioja Soria.

[72] Gray CL, Berry L (2017). Cranial nerve mischief masquerading as food allergy: auriculotemporal (Frey’s) syndrome in a child with Moebius syndrome. Curr Allergy Clin Immunol.

[73] Shah JS, Asrani VK (2017). Post herpetic Frey’s syndrome. Ann Maxillofac Surg.

[74] Williams N, Patel N, Khakoo A (2017) Frey’s syndrome as a differential diagnosis for food allergy: a case report. BJGP Open 1:bjgpopen17X100653 10.3399/bjgpopen17X100653 PMC6172667 30564644

[75] Guri A, Scheier E, Garty Y (2018) Frey syndrome following herpes zoster in an otherwise healthy girl. BMJ Case Rep 2018:bcr2018224548 10.1136/bcr-2018-224548 PMC6067137 30049675

[76] Henry N, Baker BG, Iyer S (2018). Frey’s syndrome following a facial burn treated with botulinum toxin. Ann Burns Fire Disasters.

[77] Ospina-Cantillo JA, Ramírez-Giraldo RH, Castelblanco-Arango IY, Cardona R (2018). Síndrome Frey en la consulta de alergología [Frey’s syndrome in the allergology consultation]. Rev Alerg Mex.

[78] Petel R (2019). Auriculotemporal nerve syndrome (Frey’s syndrome): a literature review and case report. J Indian Soc Pedod Prev Dent.

[79] Aragonés Redó M, Martínez Beneyto P, Marco AJ (2020). Frey syndrome in a child with neurofibromatosis type 1. Acta Otorrinolaringol Esp (Engl Ed).

[80] Kurian SR, Siegfried EC (2020). Frey syndrome-like developmental dysautonomia in a child with PHACE syndrome. Pediatr Dermatol.

[81] Smith A, Jonas N (2020). Frey’s syndrome. N Engl J Med.

[82] Ullah F, Tien M (2021). Recurring facial rash after eating in a 12-month-old girl. Pediatr Rev.

[83] O’Grady JP, Pope CS, Hoffman DE (2002). Forceps delivery. Best Pract Res Clin Obstet Gynaecol.

[84] Mansoor DK, Sharma HP. Wang LJ, Mu SC, Lin MI, Sung TC, Chiang BL, Lin CH (2022) Clinical manifestations of pediatric food allergy: a contemporary review. Clin Rev Allergy Immunol 62:180–199 10.1007/s12016-021-08895-w 34519995

[85] Blanc S, Bourrier T, Boralevi F, Sabouraud-Leclerc D, Pham-Thi N, Couderc L, Deschildre A, Dutau G, Albertini M, Tran A, Giovannini-Chami L (2016). Frey syndrome collaborators. Frey Syndrome J Pediatr.

[86] Lau BW (1989). Reassurance does not always help. Can Fam Physician.

[87] Kathol RG (1997). Reassurance therapy: what to say to symptomatic patients with benign or non-existent medical disease. Int J Psychiatry Med.

